# Probing the Nanoscopic Thermodynamic Fingerprint of Paramagnetic Ligands Interacting with Amphiphilic Macromolecules

**DOI:** 10.3390/polym9080324

**Published:** 2017-07-31

**Authors:** Jörg Reichenwallner, Christian Schwieger, Dariush Hinderberger

**Affiliations:** Institute of Chemistry, Martin-Luther-Universität Halle-Wittenberg, Von-Danckelmann-Platz 4, 06120 Halle, Germany; joerg.reichenwallner@chemie.uni-halle.de (J.R.); christian.schwieger@chemie.uni-halle.de (C.S.)

**Keywords:** amphiphilic polymers, core-shell polymers, ligand binding, ESR/EPR spectroscopy, binding thermodynamics

## Abstract

Self-assembly of macromolecules with ligands is an intricate dynamic process that depends on a wide variety of parameters and forms the basis of many essential biological processes. We elucidate the underlying energetic processes of self-assembly in a model system consisting of amphiphilic core-shell polymers interacting with paramagnetic, amphiphilic ligand molecules from temperature-dependent continuous wave electron paramagnetic resonance (CW EPR) spectroscopy subsequent to spectral simulation. The involved processes as observed from the ligands’ point of view are either based on temperature-dependent association constants (*K*_A,*j*,*k*_) or dynamic rotational regime interconversion (IC) constants (*K*_IC,*j*,*k*_). The interconversion process describes a transition from Brownian (*b*_1_) towards free (*b*_2_) diffusion of ligand. Both processes exhibit non-linear van’t Hoff (ln*K* vs. *T*^−1^) plots in the temperature range of liquid water and we retrieve decisive dynamic information of the system from the energetic fingerprints of ligands on the nanoscale, especially from the temperature-dependent interconversion heat capacity (∆*C*°_P,IC_).

## 1. Introduction

**Figure 1:**
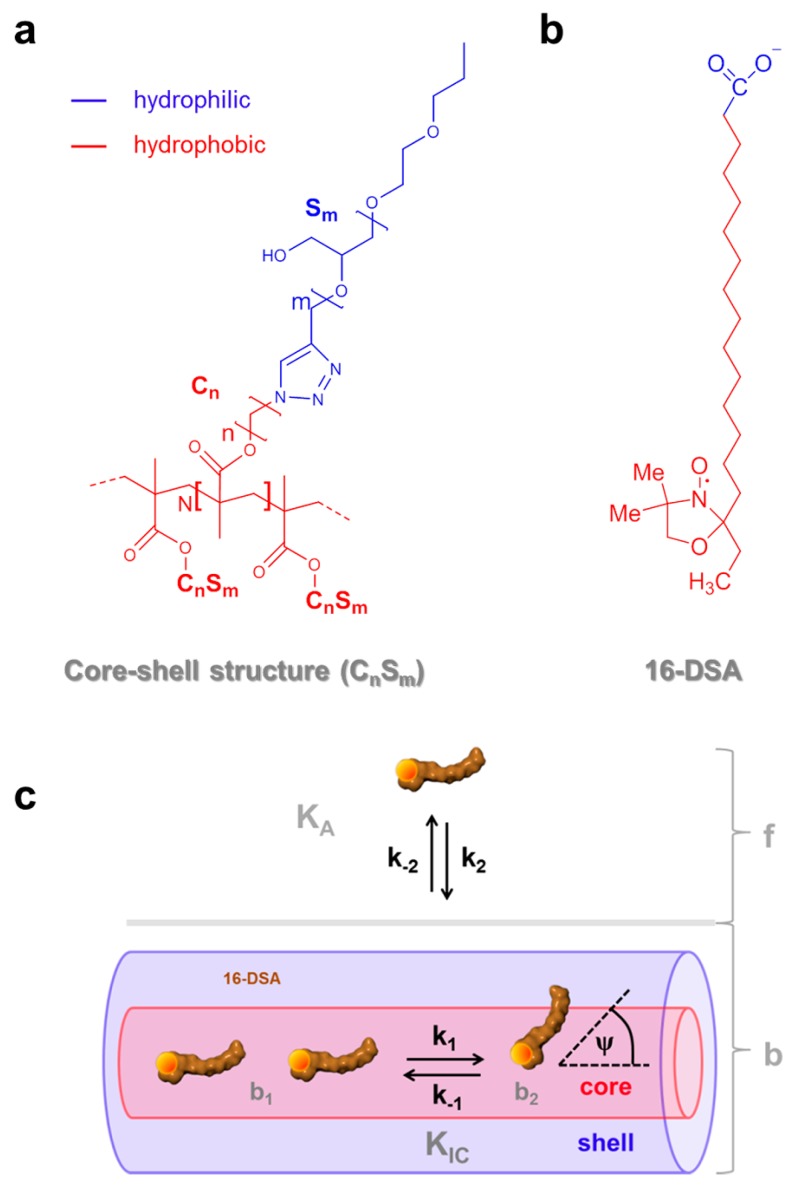
Chemical structure of the amphiphilic self-organizing components and essential dynamic regimes of 16-DSA. (**a**) Amphiphilic core-shell polymers C_n_S_m_ with degree of polymerization *N* and (**b**) the amphiphilic 16-DSA (16-doxyl stearic acid) spin probe bearing a paramagnetic nitroxide moiety (NO^•^) with color coded hydrophobic (red) and hydrophilic (blue) topological regions; (**c**) Schematic model for dynamic hydrophobic binding (*K*_A_) and dynamic hydrophobic interconversion (*K*_IC_) of 16-DSA in free (*f*) and bound (*b* comprising *b*_1_ and *b*_2_) rotational regimes in core-shell-polymers adopted from ref. [23]. The symbols *k*_i_ denote two independent processes with rate constants *k*_1_ and *k*_2_, whereas *ψ* is the spontaneous diffusion-tilt angle of the 16-DSA molecular axis in the so-called free diffusion process [24,25].

Thermodynamic profiles of macromolecules are nowadays routinely obtained with calorimetric methods. While differential scanning calorimetry (DSC) directly monitors phase transition temperatures (*T*_m_) [1] and molar heat capacity changes (Δ*C*_P_) [2], isothermal titration calorimetry (ITC) additionally delivers a quantitative account of interactions in solution, such as small molecule binding to macromolecules with their corresponding binding stoichiometry (*N*), association constants (*K*_A_) and molar enthalpy changes (Δ*H*) [3]. Depending on the macromolecular system the resulting thermodynamic quantities (Δ*H*, Δ*S* = molar entropy changes, Δ*G* = molar free energy changes, Δ*C*_P_) may also be explicitly temperature-dependent. In case a physically or chemically induced phase transition of a macromolecule occurs, e.g., from a folded to an unfolded state or a dimerization, the corresponding van’t Hoff plot, i.e., a graph of ln*K* plotted versus reciprocal temperature *T*^−1^, exhibits an inflection point representing the midpoint transition temperature *T*_m_ of the involved process. Otherwise, the van’t Hoff plot will be linear [4]. The observation of non-linear van’t Hoff plots was first reported by Brandts [5] and several other groups [6,7], subsequently. Several strategies have been developed to extract thermodynamic parameters from curves deviating from linearity. Although being considered as an exotic analytical method, thermodynamic calorimetry [8] can be used to obtain thermodynamic data from any physico-chemical (here spectroscopic) approach in which heats of reaction cannot be measured directly. One only needs the ability to simultaneously record and distinguish two different dynamic, temperature-dependent states of a macromolecular system that are linked by an equilibrium constant *K*. The most common approach for quantitative evaluations of non-linear van’t Hoff plots comprises the application of second [5,9,10] or higher order [4,11] polynomials for modeling the temperature dependence of ln*K* as a function of *T*^−1^ and the general subsequent calculation of changes in molar enthalpy (∆*H*), molar entropy (∆*S*) and molar heat capacity (∆*C*_P_). In the last decades, this method has been successfully applied in investigations of proteins and was adopted for methods such as hydrophobic interaction chromatography (HIC) [9], reversed-phase high performance liquid chromatography (RP-HPLC) [4,12], CW EPR [13], pulsed EPR as double electron–electron resonance (DEER) [14] and also calorimetric methods as DSC [11,15,16] and ITC [17,18,19]. The physical reason for this non-linear temperature dependence of Δ*H* is ascribed to large molar heat capacity changes Δ*C*_P_ mainly originating from a change in buried nonpolar surface area as a hallmark of the hydrophobic effect [20] and a change in internal vibrational modes [2]. At neutral pH, electrostatic interactions, e.g., in proteins, are usually weak compared to the hydrophobic effect [21]. In our study, we deal with amphiphilic core-shell polymers [22] with a hydrophilic polyglycerol shell (S_m_), and a hydrophobic alkylene core (C_n_), with indices *m* indicating the number of glycerol monomers and *n* being the number of methylene groups of the alkylene chain, respectively (Figure 1a). In these systems electrostatic interactions can be considered negligible.

Synthesis of various core-shell structures of this kind and associated EPR-spectroscopic details have already been presented in previous publications [23,26]. In water, this system may be conceptually reduced to mainly consist of dipolar H-bonding and hydrophobic interactions between ligand, water and substrate as an appropriate model that emulates hydrophobic binding to more complex polypeptide chains. The molecular basis of such dynamic intra- and intermolecular amphiphilic assemblies is not well understood for large and complex systems. Therefore, we provide an example that may aid in overcoming the lack of appropriate systems that facilitate a simplified functional view [27]. In our previous EPR study, we reported astonishing tunable *dynamic hydrophobic attachment* properties of stearic acid derivatives (16-DSA, Figure 1b) to core-shell polymers C_6_S_32_ and C_11_S_14_. The association constant *K*_A_ is derived from the relative fractions *φ_i,j,k_* of purely free (*f*) and bound (*b*) ligands (Figure 1c) as it was presented similarly by Flewelling et al. [28]. Particularly, the observed interconversion equilibrium constant *K*_IC_ (initially termed *K*_AB_ in ref. [23]) that describes the proposed temperature-induced dynamic switching from Brownian (*b*_1_) to free (*b*_2_) diffusion of bound 16-DSA molecules, appears to contain nanoscopic information on slight changes in polymer dynamics that will be analyzed thoroughly in this study. In contrast to conventional Brownian rotational diffusion, free rotational diffusion in EPR spectroscopy was defined for spontaneous reorientations of molecular axes by consecutive arbitrary angles *ψ* [24,25,29]. These different immobilized modes of diffusion are usually defined by the microscopic order-macroscopic disorder (MOMD) model [30]. Both rotational regimes could be distinguished from rigorous simulations of EPR spectra revealing a difference in intrinsic rotational correlation times τ_c_ by a scaling factor of about 2.4–3.4 [23], which is quite close to the theoretical value of 7^1/2^ ≈ 2.65 calculated for the two types of rotational motions. In ref. [23] we described (i) the just mentioned dynamic switching or interconversion from Brownian to free diffusion (*K*_IC_) of 16-DSA from spectral deconvolution to fractions *b*_1_, *b*_2_ and *f* in CW EPR, (ii) the stealth effect of the hydrophilic shell (S_m_), as well as its spatial hydrophilic collapse and (iii) partial aggregation of polymers as obtained from dynamic light scattering (DLS) results, (iv) adjustability of the dissociation constant (*K*_D,*k*_) and (v) the tunable number of binding sites or binding regions per polymer (*N*_L,*k*_). 

Several van’t Hoff approaches were applied in EPR to describe dynamic interconversion-like processes of paramagnetic moieties in proteins (linear) [31,32], as well as in order parameters (*S*) from membrane-bound lipid spin probes (non-linear) [13,33,34], but to the best of our knowledge there is no reported study analyzing non-linear van’t Hoff plots to investigate interconversion processes of bound ligand states to individual macromolecules. So, beyond the specific characterization of the dynamic hydrophobic attachment of amphiphilic molecules to the core-shell polymers we here provide a guideline for employing spin probing and EPR spectroscopy to macromolecules for complex nanoscopic thermodynamic analyses. 

Moreover, EPR spectroscopy simultaneously resolves different physical properties of the ensemble, monitoring e.g., polarity and rotational diffusion from small ligand molecules attached to or expelled from macromolecules at the nanoscopic scale. This facilitates the observation and discrimination of intrinsic motions and their changes, especially upon induction of external physical or chemical stimuli [13]. Furthermore, this study offers a general treatise concentrating exclusively on ligand binding thermodynamics while proving an unconventional strategy to advance towards an EPR-based quantification of the physical driving forces of ligand binding to macromolecules. 

## 2. Materials and Methods 

### 2.1. Materials

Synthesis of the core-shell structures presented here and associated EPR-spectroscopic details have already been presented in previous publications [23,26].

### 2.2. Methods

#### 2.2.1. EPR Spectroscopy

**Figure 2:**
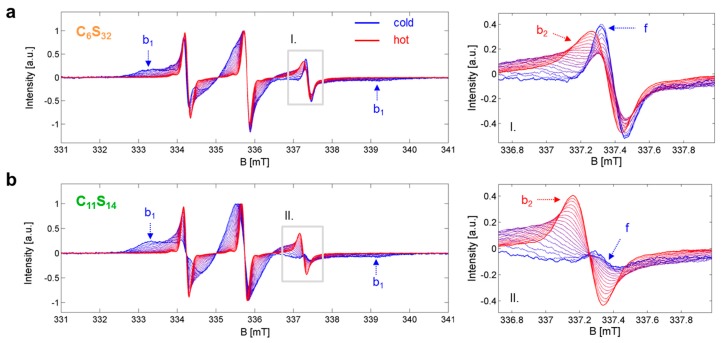
Temperature-dependent EPR spectroscopic datasets *S_j,k_*(*B*). (**a**) Core-shell polymer C_6_S_32_ and (**b**) Core-shell polymer C_11_S_14_ with the most prominent spectral features highlighted from dynamic fractions *f*, *b*_1_ and *b*_2_. The gray insets (I. in (**a**) and II. in (**b**)) on the left hand side are magnified on the right. EPR spectra were recorded [23] in the temperature range of 5 to 95 °C in steps of 5 K and the lowest (dark blue) and highest (dark red) temperature curve is depicted bold to create an envelope effect.

Sample preparation, EPR data collection (Figure 2) and strategies for spectral simulations have already been explicitly described and discussed in ref. [23]. The most important findings being also essential for this continuative study are given in Table 1. Datasets that allow the construction of *K*_A,*j*,k_ and *K*_IC,*j*,*k*_ are given in Supplementary Tables S1–S4 so that the reader may completely recalculate our findings. 

#### 2.2.2. Thermodynamic Analysis of EPR Data

**Figure 3:**
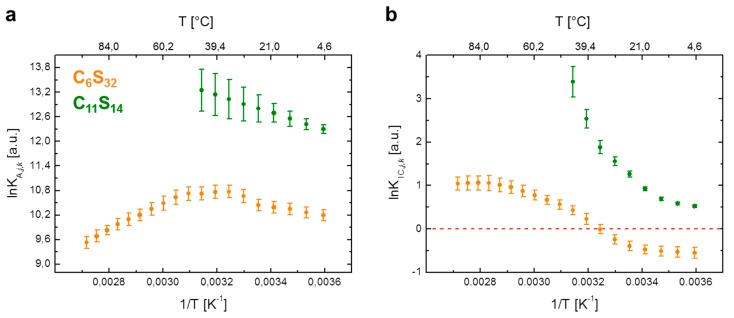
Van’t Hoff plots of 16-DSA in polymers C_6_S_32_ and C_11_S_14_ from EPR spectroscopy. Individual data points are calculated from EPR spectral deconvolution in Ref. [23] for (**a**) the ligand association constant (ln*K*_A,*j*,*k*_) and (**b**) the interconversion process between Brownian and free diffusion (ln*K*_IC,*j*,*k*_). The C_6_S_32_ polymer curves are represented in orange and C_11_S_14_ polymer curves in green throughout the whole manuscript. For raw data see Supplementary Tables S2 and S4.

**Figure 4:**
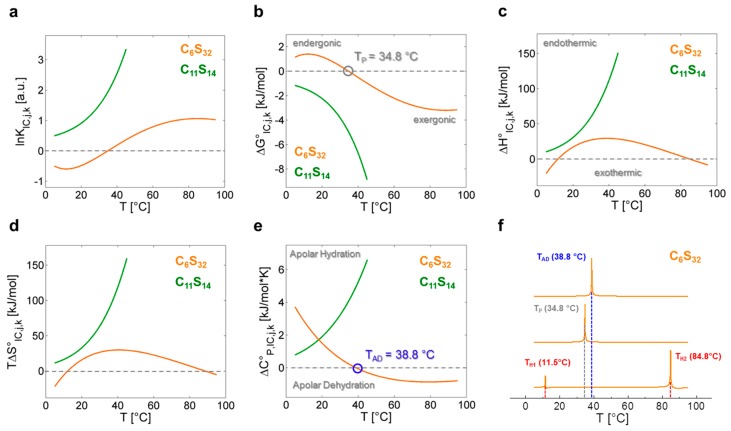
Graphical representation of the thermodynamic functions calculated from ln*K*_IC,*j,*k_. Continuous depiction of (**a**) ln*K*_IC,*j*,*k*_ vs. *T* from data in Supplementary Tables S2 and S4, Equations (12) and (16), resulting in the (**b**) molar Gibb’s free energy change ∆*G°*_IC,*j*,*k*_ with performance temperature *T*_P_ at 34.8 °C (gray), endergonic (∆*G°*_IC_ > 0) and exergonic (∆*G°*_IC_ < 0) regions; (**c**) Temperature- dependent change of molar enthalpy ∆*H°*_IC,*j*,*k*_ calculated from Equations (13) and (17) with endothermic (∆*H°*_IC_ > 0) and exothermic (∆*H°*_IC_ < 0) regions; (**d**) Temperature-dependent change of molar entropy *T*∆*S°*_IC,*j*,*k*_ calculated from Equations (14) and (18); (**e**) Change of molar heat capacity ∆*C°*_P,IC,*j*,*k*_ with the apolar dehydration temperature *T*_AD_ = 38.8 °C (blue) highlighted, as calculated from Equations (15) and (19). Regions of apolar hydration (∆*C°*_P,IC_ > 0) and apolar dehydration (∆*C°*_P,IC_ < 0); (**f**) Determination of *T*_P_, *T*_AD_ and compensation temperatures [4] *T*_H1_ and *T*_H2_ (red) for polymer C_6_S_32_ using the normalized absolute values of the functional ratios [44] |∆*S°*_IC,*j*,6_/∆*G°*_IC,*j*,6_|, |∆*S°*_IC,*j*,6_/∆*H°*_IC,*j*,6_| and |∆*H°*_IC,*j*,6_/∆*C°*_P,IC,*j*,6_| as denoted in Equations (20)–(22). In all figures C_6_S_32_ polymer curves are represented in orange and C_11_S_14_ polymer curves are in green.

All calculations of thermodynamic quantities emerging from equilibrium constants *K*_A,*j*,k_ (Figure 3a) and *K*_IC,*j*,*k*_ (Figure 3b) have been facilitated with fit parameters obtained with linear, polynomial and exponential curve regression methods in Microcal Origin 8 (Supplementary Figure S1 and Supplementary Tables S5 and S6). Thermodynamic functions for ln*K*_IC,*j*,*k*_, Δ*G°*_IC,*j*,*k*_, Δ*H°*_IC,*j*,*k*_, Δ*S°*_IC,*j*,*k*_ and Δ*C°*_P,IC,*j*,*k*_ as shown in Figure 4 were computed in a quasi-continuous 500 point grid corresponding to a 0.08–0.18 K temperature resolution. This was done with homewritten codes in MATLAB R2008b (v7.7, The MathWorks, Inc., Natick, MA, USA). Therein, the fit parameters from the aforementioned curve regressions in Origin were incorporated. 

#### 2.2.3. DSC Measurements

Differential Scanning Calorimetry (DSC) was performed with a Microcal VP-DSC (MicroCal Inc., Northampton, MA, USA). In all experiments we used a heating rate of 0.5 K/min and a cooling rate of 1 K/min. Data were recorded with a time resolution of 4 s in the temperature range of 5–95 °C, the same temperature range as for previous EPR experiments [23]. Three consecutive up- and down scans were performed for each sample to allow equilibration and to prove the reproducibility. The presented curves originate from the second heating scan (Supplementary Figure S2). The third heating scan was identical to the second one. C_6_S_32_/16-DSA and C_11_S_14_/16-DSA suspensions were prepared as for the EPR experiments and loaded to the sample cell. The concentration of macromonomers was 1.66 and 1.78 mM and the 16-DSA concentration was 29.1 and 18.4 µM for C_6_S_32_/16-DSA and C_11_S_14_/16-DSA, respectively. In addition, 16-DSA was measured without polymers below its critical micellization concentration (CMC) at pH 8.0–9.5 (KOH). The reference cell was filled with degassed ultrapure water. From all presented polymer/16-DSA thermograms a water/water reference as well as a thermogram of pure 16-DSA was subtracted, before normalizing Δ*C*_P_ to the macrounimer concentrations. Data processing was performed with the DSC module for ORIGIN software supplied by MicroCal Inc., Northampton, MA, USA.

## 3. Results

### 3.1. Thermodynamic Considerations for 16-DSA Ligand Binding to C_n_S_m_ Core-Shell Polymers

The basis for our analytic approach is the presence of thermodynamic equilibria between distinct dynamic states in the ligand ensemble that have separable EPR spectroscopic features representing the corresponding microenvironments. In particular, the association constant *K*_A_ and the interconversion constant *K*_IC_ values can be assessed by the different fractions *φ_i,j,k_* of free (*f*) and bound (*b*_1_, *b*_2_) dynamic regimes of 16-DSA interacting in a temperature-dependent manner with the polymers as shown in Figure 2. Where appropriate, temperatures are from now on abbreviated by index *j* in °C, dynamic regimes by index *i* and polymers by index *k* (*k* = 6 for C_6_S_32_ and *k* = 11 for C_11_S_14_). The resulting multicomponent EPR spectra can be thoroughly analyzed using spectral simulations as it has already been discussed extensively in ref. [23]. For example, insets I and II in Figure 2 show how the free spectral component *f* vanishes with rising temperature in favor of an ever growing *b*_2_ component, while simultaneously the *b*_1_ component vanishes noticeably with increasing temperature. Thus, we are able to observe the two different dynamic processes from the ligands’ point of view: either being free or bound (*K*_A,*j*,*k*_ from *φ_f,j,k_* and *φ_b,j,k_*) and also being in the Brownian (*b*_1_) or in the free diffusion regime (*b*_2_) (*K*_IC,*j*,*k*_ is obtained from *φ_b_*_1*,j,k*_ and *φ_b_*_2*,j,k*_) from each temperature-dependent EPR spectrum *S_j_*_,*k*_(*B*).

For our thermodynamic analysis, it is therefore inevitable to develop simple kinetic models for the quantitative description of the strong temperature dependences of 16-DSA association and interconversion of rotational diffusion regimes while bound to the C_6_S_32_ and C_11_S_14_ polymers. First, we propose a general kinetic model for the interconversion process from Brownian (*b*_1_) to free diffusion (*b*_2_) with the rate equation:

(1) $$b_{1}\overset{k_{1,j,k}}{\underset{k_{-1,j,k}}{⇌}}b_{2}$$

while assuming a temperature-dependent equilibrium of forward (*k*_1,*j*,*k*_) and backward reaction (*k*_–1,*j*,*k*_) at each temperature *j* corresponding to an equilibrium constant *K*_IC,*j,k*_ that is defined as:

(2) $$K_{\mathrm{IC},j,k}=\frac{k_{1,j,k}}{k_{-1,j,k}}=\frac{\phi_{b2,j,k}}{\phi_{b1,j,k}}=\frac{\phi_{b2,j,k}}{\left(1-\phi_{b2,j,k}\right)}=\frac{{\left[L\right]}_{b2,j,k}}{{\left[L\right]}_{b1,j,k}}$$

with *φ_b,j,k_* being the temperature-dependent spectral fraction *i* of each bound component *b*_1_ *or b*_2_ and [L]*_b,j,k_* is the corresponding concentration of ligand in the two bound regimes. Secondly, the convenient binding of a ligand L itself to a hypothetical arbitrary binding site or receptor R gives the receptor-ligand-complex RL by the relation [35]:

(3) $$R+L\overset{k_{2,j,k}}{\underset{k_{-2,j,k}}{⇌}}\mathrm{RL}$$

where *k*_2,*j*,*k*_ is the association rate constant and *k*_–2,*j*,*k*_ is the dissociation rate constant. Due to the mass action law the equilibrium association constant *K*_A,*j*,*k*_ is given by:

(4) $$K_{A,j,k}=\frac{{\left[RL\right]}_{j,k}}{{\left[R\right]}_{f,j,k}{\left[L\right]}_{f,j,k}}$$

with [R]*_f,j,k_* being the free receptor concentration, [L]*_f,j,k_* the free ligand concentration and [RL]*_j_*_,*k*_ the concentration of the receptor-ligand-complex, all of which can also be substituted by the fractions *φ_i,j,k_* of each spectral component. [RL]*_j_*_,*k*_ may simply be replaced by the bound fraction of ligand [L]*_b,j,k_* = [L]*_b_*_1,*j*,*k*_ + [L]*_b_*_2,*j*,*k*_ that is directly accessible from spectral deconvolution (Supplementary Tables S1 and S3). As we encounter tight binding characteristics with a total ligand concentration [L]*_t,k_* that by far exceeds the free ligand concentration [L]*_f,j,k_* in our self-organized systems ([L]*_f,j,k_* << [L]*_t,k_*), Equation (4) must be rewritten as [35]:

(5) $$K_{A,j,k}=\frac{{\left[\mathrm{RL}\right]}_{j,k}}{{\left([R\right]}_{t,k}-{\left[\mathrm{RL}\right]}_{j,k})\cdot {\left([L\right]}_{t,k}-{\left[\mathrm{RL}\right]}_{j,k})}$$

where [R]*_t,k_* is the total receptor or binding site concentration. After several rearrangements expressing the polymer concentration *c*_P,*k*_ and the receptor number *N*_L,*k*_ (Table 1) in terms of total receptor concentration [R]*_t,k_* = *c*_P,*k*_·*N*_L,*k*_, it can be shown that a simple and practical formula emerges that contains only terms that are experimentally accessible from EPR spectroscopy and sample preparation:

(6) $$K_{A,j,k}={\left[{\left[R\right]}_{t,k}\left(\frac{{\left[L\right]}_{t,k}}{{\left[L\right]}_{b,j,k}}-1\right)-{\left[L\right]}_{f,j,k}\right]}^{-1}$$

An explicit derivation of Equation (6) is given in the Supplementary Information (Equation (S9)). The individual values that have been used for our analysis are given in Table 1 and Supplementary Tables S1–S4 and are taken from previous studies [23,26]. The values for *c*_P,*k*_ and *N*_L*,k*_ are assumed to remain largely constant over the whole observed temperature range as they predominantly dependent on the degree of polymerization *N* [23]. 

First, we study the temperature dependence of the association constant *K*_A,*j*,*k*_ in a van’t Hoff plot, i.e., by plotting ln*K*_A,*j*,*k*_ against inverse temperature in Figure 3a. For the C_6_S_32_ polymer, the non-linear van’t Hoff plot can be significantly simplified by subdividing the process into separate temperature regimes and inspecting the two regimes between 5–25 °C and 55–95 °C first, where the curve practically resembles straight lines (orange curve). Between 30–50 °C, the association constant remains at a value of *K*_A,*j*,6,max_ = (4.6 ± 0.2) × 10^4^·M^−1^ that corresponds to a dissociation constant of *K*_D,*j,*6,min_ = (21.9 ± 0.1) µM (Supplementary Table S2).

However, in direct comparison the C_11_S_14_ polymer remarkably shows a straight decrease of ln*K*_A,*j*,*k*_ in the van’t Hoff plot between 5 to 45 °C (Figure 3a). In this context, the method of choice for a quantitative analysis of van’t Hoff plots is the well-known linear extrapolation of data points, as the principles of ligand binding energetics are described by [36]:

(7) $$\Delta G_{A,j,k}^{\circ }=-RT\ln K_{A,j,k}=RT\ln K_{D,j,k}$$

where *K*_A,*j*,*k*_ and *K*_D,*j*,*k*_ are the binding association and dissociation constants (*K*_A,*j*,*k*_ = *K*_D,*j*,*k*_^−1^) [35], *R* is the universal gas constant and *T* is here the absolute temperature in Kelvin.

We can now combine Equation (6) with Equation (7) to get an expression for ln*K*_A,*j*,*k*_ as obtained from our EPR data:

(8) $$\begin{array}{ll} \ln K_{A,j,k} & =-\ln\left[{\left[R\right]}_{t,k}\left(\frac{{\left[L\right]}_{t,k}}{{\left[L\right]}_{b,j,k}}-1\right)-{\left[L\right]}_{f,j,k}\right]\\ & =-\frac{\Delta G_{A,j,k}^{\circ }}{RT}=\frac{\Delta S_{A,j,k}^{\circ }}{R}-\frac{\Delta H_{A,j,k}^{\circ }}{R}\cdot \frac{1}{T} \end{array}$$

Here, ∆*H°*_A,*j*,*k*_/*R* is the slope and ∆*S°*_A,*j*,*k*_/*R* is the *y*-axis intercept of the straight line comprising the standard molar enthalpy (∆*H°*_A,*j*,*k*_) and entropy changes (∆*S°*_A,*j*,*k*_) of the ligand association process (see also Equation (S10)). The results from this analysis are shown in Table 2 and Supplementary Table S5.

Linear fits to the experimental ln*K*_A,*j*,*k*_ curves with an *R*^2^ between 0.9841 and 0.9989 are shown in Supplementary Figure S1a–c. Apparently, 16-DSA association at low temperatures (*T* < 30 °C) is reproduced by a line with negative slope for both polymers indicating a weakly endothermic binding process (∆*H°*_A,25,*k*_ > 0:8.6 kJ·mol^−1^ for C_6_S_32_ and 17.5 kJ·mol^−1^ for C_11_S_14_) with high positive entropy changes of 115.7 J·mol^−1^·K^−1^ for the C_6_S_32_ polymer and 165.2 J·mol^−1^·K^−1^ for the C_11_S_14_ polymer (∆*S°*_A,25,*k*_ > 0).

Hence, this is an exergonic (Δ*G°*_A,25,*k*_ < 0) and an entropy driven [12] (Δ*H°*_A,25,*k*_ > 0, Δ*S°*_A,25,*k*_ > 0) reaction in the investigated low temperature ranges (see Table 2). From our data we interpret that the longer the alkylene spacer C_n_ and the more nonpolar the hydrophobic core (*a*_iso,*j*,*k*_, Table 1), the more positive the entropy changes and the more endothermic the ligand association process become, where thermal energy from the environment is converted into binding energy. 

While linearity is found throughout the whole observable temperature range for C_11_S_14_, there is a slope inversion between 30 °C < *T* < 50 °C for C_6_S_32_. For *T* > 50 °C the ligand binding process is converted into a comparatively strong exothermic process (∆*H°*_A,>50,6_ = −27.1 kJ·mol^−1^) with a much smaller entropy increase upon ligand association of ∆*S°*_A,>50,6_ = 5.8 J·mol^−1^·K^−1^ than for low temperatures (∆*S°*_A,25,6_ = 115.7 J·mol^−1^·K^−1^). So, this high temperature decrease in ligand affinity can still be regarded as exergonic in terms of ligand binding (Δ*G°*_A,>50,6_ < 0) and was shown to be correlated with partial aggregation of C_6_S_32_ polymers for *T* > 35 °C (Table 1). From DLS measurements and corresponding −log *τ*_c,*j*,*k*_ curves from EPR data [23] we identified a partial hydrophobic polymer aggregation induced by a dynamic change in the C_6_S_32_ polymer–water interaction between 30 °C < *T* < 40 °C (HCT, Table 1). This change was finally assigned to the collapse of the hydrophilic shell. We conclude that this partial aggregation subsequent to the hydrophilic shell collapse leads to a reduced fatty acid binding affinity with increasing temperature due to sterical hindrance for ligand binding, accompanied by a consequent strong negative enthalpy change (Δ*H°*_A,>50,6_ < 0). Hence, the C_11_S_14_ polymer provides a higher binding affinity *K*_A_ and entropy gain for *T* < 35 °C (∆*S°*_A,25,11_ > ∆*S°*_A,25,6_, Table 1) and therefore ligand binding to the hydrophobic core-region is apparently enhanced by the absence of aggregation in direct comparison to the C_6_S_32_ polymer [23]. The strategy of extracting *K*_A_ values is nowadays routinely applied in EPR spectroscopy [37,38,39,40,41] corresponding to findings that can be made from ITC studies. Furthermore, in our study we refrain from applying ITC as the solution properties may change decisively in chemical potential, pH, and ionic strength upon 16-DSA ligand titration that is typically dissolved in potassium hydroxide (KOH). Furthermore, the next section will expand this treatise to an analytic method that is assumed to be exclusively accessible by EPR spectroscopy.

Regarding the temperature-dependent rotational diffusion regime interconversion constant *K*_IC,*j*,*k*_ as shown in Figure 3b, a more intricate approach is required compared with the ligand association process. The aim is to introduce fit functions for ln*K*_IC,*j*,*k*_ to reproduce the obtained van’t Hoff curve shapes [4] as it was recently shown for gel and fluid-like phases in pulmonary surfactants by EPR [13]. These fit curves then facilitate the derivation of the required thermodynamic functions (Δ*H*, Δ*S*, etc.). For the ln*K*_IC,*j*,*k*_ -plot in Figure 3b of the C_6_S_32_ and C_11_S_14_ polymers we henceforth observe non-linearity for both polymers. Basically, the temperature dependence of equilibrium constants *K_i_* is usually well described by the practical form of the van’t Hoff equation [42,43].

(9) $$\frac{d\ln K_{i}}{d\left(\frac{1}{T}\right)}=-\frac{\Delta H_{v.H.}}{R}$$

The interconversion processes in Figure 3b can therefore be thermodynamically evaluated with a continuative set of equations [9,17]:

(10) $$\Delta G_{\mathrm{IC},j,k}^{\circ }=-RT\ln K_{\mathrm{IC},j,k}^{}=\Delta H_{\mathrm{IC},j,k}^{\circ }-T\Delta S_{\mathrm{IC},j,k}^{\circ }$$

(11) $$\Delta C_{P,\mathrm{IC},j,k}^{\circ }={\left(\frac{\partial \Delta H_{\mathrm{IC},j,k}^{\circ }}{\partial T}\right)}_{P}$$

The fit functions for the curves of ln*K*_IC,*j*,*k*_ vs. *T*^−1^ for C_6_S_32_ and C_11_S_14_ polymers that have been found to describe the curve progressions best are summarized in Table 3. While the C_6_S_32_-polymer requires the application of a fourth order polynomial (Equation (12)), in case of C_11_S_14_ it suffices to use a simple exponential function (Equation (16)) although not conventionally applied to such kind of analysis. Equations (13)–(15) and (17)–(19) in Table 3 are then derived from Equations (12) and (16) by applying the mathematical expressions in Equations (9)–(11). Both original non-linear fit curves of data shown in Figure 3b are given in Supplementary Figure S1d,e. 

In Figure 4 all fit-derived functions of Table 3 are plotted for both polymers in the whole temperature range investigated. For a polynomial analysis of the C_6_S_32_ polymer the fit parameters are α*_x_* (*x* = 1–5), while the fit parameters for the exponential analysis of the C_11_S_14_ polymer are denoted as *κ_y_* (*y* = 1–3). A complete set of fit parameters is given in Supplementary Table S6. From the functions for ln*K*_IC,*j*,*k*_ (Figure 4a), the free energy changes ∆*G*°_IC,*j*,*k*_ of the interconversion process (Figure 4b) are obtained in a straightforward procedure from the well-known relation ∆*G*°_IC,*j*,*k*_ = −*RT*ln*K*_IC,*j*,*k*_ in compliance with Equation (7). It is reasonable to assume that the interconversion process of dynamic ligand regimes may reflect a structural and/or dynamic transition in the combined ligand–polymer systems. Hence, the system is in a state in which small structural perturbations may lead to changes in stability and therefore to small changes in the melting temperatures *T*_m_ = Δ*H°·*Δ*S°^−^*^1^ where free energy is zero (∆*G°* = 0) [4,44], as it has been extensively discussed for proteins [45]. In the following, we aim at tracking these dynamic instabilities of the rotational regime interconversion *K*_IC_ with the described strategies from protein biophysics [8]. This aspect of our analysis facilitates access to thermodynamic properties of polymer-bound ligands from a nanoscopic view on the system without measuring macroscopic properties like heat. 

As the C_11_S_14_ polymer shows exponentially shaped curves for ln*K*_IC,*j*,11_, ∆*G°*_IC,*j*,11_, ∆*H°*_IC,*j*,11_, ∆*S°*_IC,*j*,11_ and ∆*C°*_P,IC,j,11_ (Figure 4a–e, green) without any zero crossings, we can assume that the interconversion process from Brownian to free diffusion (*K*_IC,*j*,11_) is purely entropy driven at all temperatures for this polymer (∆*H°*_IC,*j*,11_ > 0, ∆*S°*_IC,*j*,11_ > 0). The rise in positive ∆*C°*_P,IC,*j*,11_ with temperature denotes increased apolar hydration (∆*C*_P_ > 0) [8] of the bound ligands illustrating the mere opening of the buried core segments from low to higher temperatures without any macroscopically detectable transitions (see below). As ligand association to this polymer can in principle be regarded as being entropy driven and exergonic at all temperatures (∆*H°*_A,*j*,11_ > 0, ∆*S°*_A,*j*,11_ > 0, ∆*G°*_A,*j*,11_ < 0; Table 2), this finding is also in agreement with our previous DLS data, as partial C_11_S_14_-aggregates were shown to vanish above 35 °C increasing the ligands’ accessibility to the hydrophobic core (Table 1). The reduction in the spectral fraction of strongly bound ligands (*b*_1_), however, goes along with a simultaneous depletion of the free fraction (*f*) of 16-DSA probes in our EPR spectra (Figure 2). Thus, the ligand binding affinity (*K*_A_), i.e., the change from the free to *b*_1_-bound ligand state does not seem to be correlated to the interconversion process (*K*_IC_). 

C_6_S_32_ exhibits a zero crossing for ln*K*_IC,*j*,6_ and ∆*G°*_IC,*j*,6_ at *T* = 34.8 °C (Figure 4a,b) when both bound dynamic fractions *b*_1_ and *b*_2_ are occupied to exactly 50% each. Interestingly, this is also the temperature region in which the amount of free ligand *f* is at its minimum and therefore *K*_A,*j*,*k*_ is at its maximum (Figure 3a). At this temperature, polymer aggregation is initiated and the hydrophilic shell collapse is most pronounced as seen in DLS data [23]. So, the hydrophilic shell of the C_6_S_32_-polymer is in its most densely packed (*R*_H,*j*,6,min_) [23], and best ligand-binding (*K*_A,*j*,6,max_) state. Therefore, this characteristic temperature is from here on called *performance temperature* at *T*_P_ = 34.8 °C. At this same temperature the bound ligands monitor a thermodynamic instability (Δ*G°*_IC,TP,6_ = 0) of the energetic landscape leading to more ligands exhibiting free diffusion at temperatures above *T*_P_. While this interconversion process is endergonic at low temperatures (∆*G°*_IC,<TP,6_ > 0) it spontaneously proceeds (∆*G°*_IC,>TP,6_ < 0, exergonic) at all temperatures above *T*_P_ with the interconversion equilibrium being then shifted towards free diffusion. From Figure 4c,d we can see that the interconversion equilibrium (*K*_IC,*j*,6_) is entropy driven between about 12 °C < *T* < 85 °C (∆*H°*_IC,12–85,6_ > 0, *T*∆*S°*_IC,12–85,6_ > 0), and enthalpy driven below and above these temperatures (∆*H°*_IC,12>*j*>85,6_ < 0, *T*∆*S°*_IC,12>*j*>85,6_ < 0). Another marked difference of bound ligands among the two polymers is the behavior of the change in molar heat capacity ∆*C°*_P,IC,*j*,*k*_ (Figure 4e) of the interconversion process. 

While C_11_S_14_ has a positive and constantly increasing heat capacity change that is indicative of apolar hydration by increased hydrophobic surface exposure of the polymer core, ∆*C°*_P,IC,*j*,6_ of C_6_S_32_ constantly decreases in the whole temperature range with a zero crossing at *T*_AD_ = 38.8 °C, the *apolar dehydration temperature*. This second characteristic temperature therefore marks the transition from a system with slight *apolar hydration* below *T*_AD_ to distinct *apolar dehydration* [8] of the ligands above *T*_AD_. This can be directly correlated to the solvent accessibility of the hydrophobic core of the polymers, where the bound paramagnetic ligand probes are located. The zero crossing of ∆*C°*_P,IC,*j*,6_ is also exactly where ∆*H°*_IC,*j*,6_ and *T*∆*S°*_IC,*j*,6_ have their individual overall maximum value (*T*_AD_ = 38.8 °C) according to Equation (11), as the Δ*C°*_P,IC,*j*,6_-curve exhibits a zero crossing where ∆*H°*_IC,*j*,6_ exhibits curve slope inversion. This result is also completely in line with the observed onset of aggregation of the C_6_S_32_ polymers from DLS data (Table 1) and gives a test of the validity of our equations. As the C_6_S_32_ polymer is not aggregated below 35 °C, the hydrophobic fatty-acid bearing core in this state is much more accessible to solvent and therefore accessible to apolar hydration. 

This inverting behavior of the sign of ∆*C°*_P,IC,*j*,6_ can be directly linked to the hydrophilic shell collapse and subsequent polymer aggregation. The endothermic, temperature-induced increase in ligand affinity of polymer C_11_S_14_, that initially also occurs for C_6_S_32_, is therefore expected to counteract the increased solvent hydration of the hydrophobic core and indirectly reflects its increased accessibility. Due to the thinner hydrophilic shell, we assume that the hydrophobic surface exposure of C_11_S_14_ to water is too high and cannot be counterbalanced by a (thick) hydrophilic shell collapse that induces hydrophobic self-assembly and aggregation [27] as for polymer C_6_S_32_.

Nanoscopically derived, thermodynamic EPR data for polymer C_6_S_32_ support the picture of a soft dynamical transition at *T*_P_ = 34.8 °C to a more compact state with a hydrophilic shell collapse at its origin. Further evidence has also been found in the slightly depleted rotational correlation times τ_c,*j*,6_ of ligands in the *b*_2_ rotational regime and reduced hydrodynamic radii *R*_H,*j*,6_ for this temperature range [23]. From an energetic point of view, it is also probable that the nature of this process is a compensatory hydrophilic collapse as we calculated an apolar dehydration process to explicitly occur above *T*_AD_ = 38.8 °C for the C_6_S_32_ polymer. Functionally, this could be the hallmark of a *stealth effect* of the hydrophilic shell for the C_6_S_32_ polymer, which may camouflage the hydrophobic core from apolar hydration above the hydrophilic collapse or rather the performance temperature (*T*_P_ = 34.8 °C).

In this picture the increase of free ligand for *T* > 50 °C can also be ascribed to an increased expulsion of ligand because of the aforementioned sterical restriction upon hydrophilic collapse and polymer aggregation. Using the fit-derived functions (Equations (12)–(19)) is perfectly suited for calculations of the thermodynamic parameters at *any* temperature. Therefore, a direct thermodynamic comparison of both polymers is summarized in Table 4 through their calculated ∆*G°*_IC,25,*k*_, ∆*H°*_IC,25,*k*_, ∆*S°*_IC,25,*k*_ and ∆*C°*_P,IC,25,*k*_ values at *T* = 25 °C. For further information about the complex origins of this energetic behavior the reader may be referred to the work of Privalov and Makhatadze [15,16]. From Table 4 we can also see that, especially in ∆*H°*_IC,25,*k*_ and ∆*S°*_IC,25,*k*_, the use of C_11_-spacers instead of C_6_-spacers leads to a doubling of the respective values, which makes the interconversion process (*K*_IC,*j*,*k*_) of 16-DSA increasingly favorable with rising alkylene chain length and temperature. This effect is also seen in the enthalpy values ∆*H°*_A,25,*k*_ of ligand association. Additionally, the zero crossing (∆*G°*_IC,TP,6_ = 0) that coincides with the optimum ligand binding properties for C_6_S_32_ at *T*_P_ = 34.8 °C requires that the fraction of the dynamic *b*_1_ species has to exceed 50% at low temperatures (*ϕ*_b1,<30,6_ > 0.5). Thus, the induction of a performance temperature *T*_P_ in solution in terms of ligand binding is related to the structurally tunable functional units (C_n_ and S_m_) of the core-shell polymers that energetically regulate the degree of structural hydration.

In Figure 4f we additionally present a strategy to identify characteristic temperatures of polymer C_6_S_32_ from our analysis according to following expressions:

(20) $$T_{P}=max\left\{\left|\Delta S_{\mathrm{IC},j,6}^{\circ }\cdot \Delta G_{\mathrm{IC},j,6}^{\circ }{}^{-1}\right|\right\}$$

(21) $$T_{\mathrm{AD}}=max\left\{\left|\Delta H_{\mathrm{IC},j,6}^{\circ }\cdot \Delta C_{P,\mathrm{IC},j,6}^{\circ }{}^{-1}\right|\right\}$$

(22) $$T_{H,i}=max\left\{\left|\Delta S_{\mathrm{IC},j,6}^{\circ }\cdot \Delta H_{\mathrm{IC},j,6}^{\circ }{}^{-1}\right|\right\}$$

denoting maxima (in Kelvin) of absolute values in between key energy function ratios. In principal, those maxima arise from the zero values of the denominator energy term. These directly provide the performance temperature *T*_P_ = 34.8 °C from the zero intercept of free energy (Δ*G°*_IC_) [44], the apolar hydration temperature *T*_AD_ = 38.8 °C from the zero intercept of Δ*C*°_P,IC_ as well as the enthalpy compensation temperatures [12] *T*_H1_ = 11.5 °C and *T*_H2_ = 84.8 °C from zero intercepts of (Δ*H*_IC_). 

### 3.2. Differential Scanning Calorimetry (DSC)—A Consistency Check

Results from several DSC experiments show that this standard method fails to detect those nanoscopic polymer details we found with EPR spectroscopy. Upon heating both polymer/16-DSA complexes, no temperature-dependent transitions of those assemblies could be detected, i.e., neither peaks (indicative for 1st order phase transitions) nor steps (indicative for 2nd order phase transitions) were observed in the heat capacity traces (Supplementary Figure S2). As the ligand content in the samples was very low (1.7 and 1.0 mol % for C_6_S_32_ and C_11_S_14_, respectively), any heat effects resulting from 16-DSA transitions would be below the detection limit of DSC. Conversely, the polymer concentration was chosen such that thermotropic phase transitions would be detectable, given that the polymer phases differ in enthalpy or heat capacity. The absence of such transitions shows that the variations in polymer self-aggregation that trigger the observed transitions in the ligand dynamics are not identical to thermodynamic phase transitions.

## 4. Discussion

**Figure 5:**
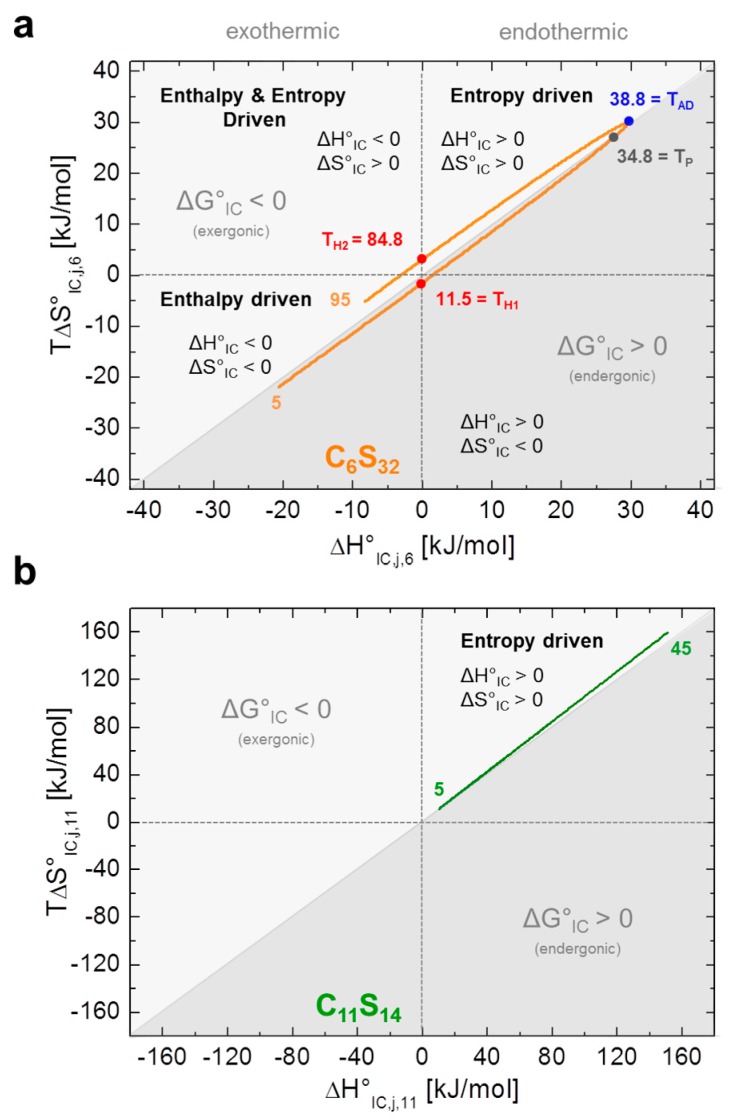
Energetic fingerprint of the interconversion process *K*_IC,*j*,*k*_. Both energetic fingerprints are depicted in the style of ref. [12]. (**a**) Energy plot of entropy (*T*∆*S°*_IC,*j*,6_) versus enthalpy (∆*H°*_IC,*j*,6_) is shown from the dynamic hydrophobic interconversion equilibrium *K*_IC,*j*,6_ of 16-DSA in polymer C_6_S_32_. The apolar dehydration temperature (*T*_AD_ = 38.8 °C) is highlighted in blue, the performance temperature (*T*_P_ = 34.8 °C) in gray and the enthalpy compensation temperatures (*T*_H1_ = 11.5 °C and *T*_H2_ = 84.8 °C) in red; (**b**) The energy plot of entropy (*T*∆*S°*_IC,*j*,11_) versus enthalpy (∆*H°*_IC,*j*,11_) of the dynamic hydrophobic interconversion process *K*_IC,*j*,11_ of 16-DSA in polymer C_11_S_14_ exhibits almost perfect EEC behavior. Exergonic and endergonic regions of the plot have been separated by a diagonal line and are color coded in different shades of gray. The orange and green inset numbers denote the respective temperatures.

We show that EPR spectroscopy on paramagnetic, amphiphilic ligands is capable to shed light on the binding behavior and some intricate thermodynamic properties of macromolecules. In case of the C_6_S_32_ polymer, striking similarities to protein-like behavior appear, as a functional stabilization and a maximum of ligand uptake (*K*_A,max_)—A temperature optimum—is indirectly monitored by an observed crossing of the fractions of the two dynamically bound components *b*_1_ and *b*_2_ of the spin probe 16-DSA (Δ*G°*_IC,Tp,6_ = 0). Energetically, the fatty acid ligands can therefore be regarded as an indirect sensor for the nanoscopic structural and dynamical agility of the polymers. They may exhibit a complicated relation between interconversion entropy (Δ*S°*_IC,*k*,6_) and enthalpy changes (Δ*H°*_IC,*k*,6_) for C_6_S_32_ or (nearly) perfect entropy–enthalpy compensation (EEC) as obtained for C_11_S_14_. This is achieved by observing and correlating their polymer bound dynamic interconversion process (*K*_IC,*j*,*k*_) with the dynamic polymer structure affecting the mode of their rotational diffusion regime (Figure 5). Linear EEC behavior is commonly observed during the unfolding process of proteins [46,47]. Here, the vanishing aggregates of C_11_S_14_ with rising temperature as monitored by DLS [23] are accompanied by an increased ligand uptake or rather an increase in ligand binding affinity due to endothermic reaction conditions. This is most probably due to the ligands’ shielding of the hydrophobic core and therefore partially preventing apolar hydration. The thermodynamic fingerprint of the polymer-bound ligands and their dynamic interconversion (*K*_IC,*j*,*k*_) is summarized in Figure 5 utilizing a well-established representation from protein biophysics [12,44], by plotting *T*Δ*S*°_IC,*j*,*k*_ versus Δ*H*°_IC,*j*,*k*_. 

From a physical methods point of view, the use of nitroxide-bearing ligands in EPR spectroscopy turns out to deliver a new perspective on the inner working of complex macromolecules and their dynamic interactions with ligands. As compared to established calorimetric methods such as isothermal titration calorimetry (ITC) or differential scanning calorimetry (DSC) this approach reveals the possibility to obtain an indirect but in-depth functional view of the polymer–ligand behavior itself without any other influences as e.g., mixing enthalpies that might alter the results or prove thermodynamical insights, e.g., into the interconversion process, inaccessible (this issue has also been pointed out in ref. [41]). The DSC measurements that have been conducted in this study turned out to be largely insensitive to effects we observed with EPR spectroscopy. Neither phase- nor glass-transitions could be detected in aqueous suspensions of the polymers. However, our previous DLS data [23] suggested a soft volume phase transition (VPT) [22,48,49] of polymer C_6_S_32_ between 30 and 40 °C that we initially termed as a hydrophilic collapse (HCT, Table 1).

Note, that the observed energetic processes from EPR (ln*K*_A,*j*,*k*_ and ln*K*_IC,*j*,*k*_) are monitored by 16-DSA and do not depict heat capacity changes (Δ*C*_P_) of the macromolecule itself. It is furthermore expected that other amphiphilic spin probes may exhibit the same or at least similar effects [41]. Our approach also has limitations, e.g., in the thermodynamic evaluation of the C_11_S_14_ polymer, as the ligand association (*K*_A,*j*,*k*_) and interconversion processes (*K*_IC,*j*,*k*_) are only detectable up to 45 °C, when the free (*f*) and the immobilized (*b*_1_) component indicating Brownian rotational diffusion vanish. Principally, the potential consequence of such data accessibility limitations is widely covered in calorimetric literature [42,50,51,52,53] that have been incorporated in this study as far as possible.

As we have shown from the van’t Hoff plot of ln*K*_A,*j*,*k*_, especially for C_11_S_14_ (Figure 3a), the fatty acid ligand 16-DSA may indeed contribute to the stability of the system consisting of polymer and ligand in water with a strong entropy increase upon binding (Table 2), while polymer aggregates dissolve and unimeric polymer structures are released [23]. For C_6_S_32_, we even observe an energetic inversion of the ligand binding process from endothermic (*T* < 30 °C) to exothermic (*T* > 50 °C) resulting from what can be described as structural breathing of the hydrophilic shell and a subsequent aggregation process [23]. It is commonly accepted that the solubility of nonpolar compounds in water, as our 16-DSA ligand, may exhibit exactly this energetic inversion behavior [54]. Additionally, the hydrophobic effect intricately depends on the length scale of the solutes [55,56] leading to contact of extended apolar surfaces of objects being less than 5 nm apart due to spontaneous water depletion. This gives a further explanation for the different aggregation behavior of both investigated polymers. The C_6_S_32_ polymer is much larger than the C_11_S_14_ polymer due to its thicker hydrophilic shell and the about 4-fold higher degree of polymerization (*N*). The combination of intermediate length hydrophobic alkylene core together with larger hydrophilic shell and large degrees of polymerization seem to make C_6_S_32_ more versatile than C_11_S_14_ in employment of the hydrophobic effect for ligand binding. Essentially, there seem to be two different functional binding modes (*b*_1_ and *b*_2_) that are only separated by rather shallow energetic and entropic barriers. 

Hydrophilic interactions play a crucial role in the amphiphilic self-assembly behavior in protein–protein association and molecular recognition [57]. It is furthermore generally assumed that the hydrophobic effect and hydrogen bonding make a comparable contribution to globular protein stability [58]. Hydrogen bonding is originally an electrostatic dipole–dipole interaction between two molecular moieties differing in electronegativity [59,60]. Principally, this leads to a broad variety of emerging dynamic and structural characteristics for macromolecules [61] that we also encounter in this study by observing temperature-induced changes of the interaction of solvent molecules with the polymers core and shell that in turn affects ligand uptake performance.

In the thermodynamic analysis of the rotational regime interconversion process, the choice of the fit functions for ln*K*_IC,*j*,*k*_ has to be made carefully, to avoid imposing unnecessary complexity into the calculation and evaluation process. The application of an exponential function for the C_11_S_14_-polymer proved to be very practical for the mathematical derivation procedures and can be analyzed in a simple manner. Analytical solutions for, e.g., calculating transition temperatures from fit-derived functions at ∆*G°* = 0 have not been found as presented in ref. [4]. We determined the values for performance temperature (*T*_P_), apolar dehydration temperature (*T*_AD_) and enthalpic compensation temperatures (*T*_H1_ and *T*_H2_) by applying the absolute values of the quotients shown in Equations (20)–(22) (Figure 4f). 

We show that by combining the alkylene cores C_n_ and hydrophilic shells S_m_ of variable length, one can structurally devise a non-trivial thermal response from the ligand interacting with these polymers and water. Although not being deliberately designed to this end, this lends them potential as an interesting and tunable [23] carrier system (C_n_S_m_) that is not necessarily restricted to stearic acid derivatives as ligands. Explicitly, this means that an increase in C_n_ spacer length strengthens the hydrophobic character of the polymer core (*a*_iso,*j*,*k*_ decreases, Table 1), the positive enthalpy changes ∆*H°*_A,25,*k*_ and also the positive entropy changes ∆*S°*_A,25,*k*_ of the system as induced by the ligand association process (*K*_A,*j*,*k*_). However, the ligand binding process remains exergonic (Δ*G°*_A,*j*,*k*_ < 0) for all investigated temperatures (Table 2). 

The hydrophilic collapse of the polyglycerol shell coupled with the polymer aggregation behavior may lead to an energetic inversion of ligand association from endothermic at low temperatures to exothermic at higher temperatures (C_6_S_32_ polymer). This energetic inversion behavior can be induced or even adjusted by a combined and targeted modification of hydrophobic spacer length C_n_, hydrophilic shell size S_m_ and the degree of polymerization *N*. 

The heat capacity signature ∆*C°*_P,IC,*j*,*k*_ of the interconversion process (*K*_IC,*j*,*k*_) can be structurally devised to switch the bound ligand state from an unfavorable apolar hydration towards an apolar dehydration (C_6_S_32_). This is derived from the polymeric self-assembly seen in previous DLS results [23] and is thermodynamically reflected in the apolar dehydration temperature *T*_AD_ = 38.8 °C where ∆*C°*_P,IC_ = 0 (Figure 4e). While polymers with long alkylene and short polyglycerol chains are subject to apolar hydration throughout the whole temperature range (C_11_S_14_), a slight stealth effect of the hydrophilic shell [23] is obtained for long polyglycerol and shorter alkylene chains, effectively shielding the hydrophobic core from apolar hydration. The inversion in direction of heat capacity change Δ*C°*_P,IC,*j*,*k*_ of the interconversion process (Figure 4e) can be regarded as facilitating the detection of a transition from a loose to a more compact structure of the polymer shell. This is accompanied by partial aggregation and an optimum performance temperature *T*_P_ = 34.8 °C for ligand uptake (*K*_A,max_) at about physiological temperatures. Therefore, the hydrophilic shell thickness S_m_ and not the core length C_n_, dominates the water accessibility of the hydrophobic core with vast effects on ligand binding and on the intricate dynamic behavior of the polymer. It could also be shown that not all combinations of C_n_ and S_m_ lead to comparable ligand binding properties [23]. Particularly, polymers with comparatively short alkylene spacer lengths (*n* < 6) do not exhibit a similar ligand binding behavior at all and constitute the observation of an interconversion process as inaccessible. 

In summary, when a slight dynamic rearrangement of the macromolecule occurs, the ligands exhibit complex entropy–enthalpy plots of *K*_IC_ whereas the absence of such a hydrophilic shell transition results in almost perfect EEC (Figure 5). Besides potential drug-delivery applications [62,63,64], we have shown that such core-shell systems appear to be well-suited to provide new experimental evidence of how the hydrophobic effect guides small molecules towards or inside transport molecules, how the mutual interaction of transport molecules with ligands occurs, and how the transport molecules may vary their functional appearance on a coarse-grained molecular level. In this study the *K*_IC_ process has been characterized in-depth and we have identified it to be useful for monitoring changes of hydration states of ligand and macromolecular substrate. This might also shed new light on various aspects in the vast research field of ligand binding to proteins or macromolecules in general. In particular, early stages of loose ligand association and conversion to more strongly bound states in e.g., ligand binding pockets and active centers of proteins may in fact be described with quantitative accuracy by this very robust, nanoscopic, EPR-based thermodynamic strategy. This study in combination with ref. [23] also outlines some predictive rules to describe self-assembly on a more structural molecular level.

## Figures and Tables

**Table 1:** Established structural and dynamic parameters of the core-shell polymers [23,26].

Method	Parameter	C_6_S_32_	C_11_S_14_
	MW_MM_^a^ (kDa)	2.72	1.46
SEC MALLS ^b,c^	MW (kDa)	470.0	64.3
	*N* ^d^	172.8	44.0
	PDI_MM_^e^	1.11	1.25
DLS ^c^	HCT ^f^ (°C)	30–40	–
	DHAT ^g^ (°C)	>35	<35
EPR ^c^	DRO ^h^	*f*, *b*_1_, *b*_2_	*f*, *b*_1_, *b*_2_
	*K*_D,25,*k*_^i^ (µM)	28.82 ± 2.57	2.42 ± 0.35
	*K*_A,25,*k*_^i^ (M^−1^)	(3.47 ± 0.31) × 10^4^	(4.13 ± 0.60) × 10^5^
	*N*_L,*k*_^j^	11.82 ± 1.37	1.96 ± 0.35
	*N*_L,*k*_·*c*_P,*k*_ (mM)	1.005	1.219
	*a*_iso,*b*_^k^ (G)	15.27	15.14
	*a*_iso,*f*_^l^ (G)	15.79	15.78

^a^ MW_MM_ = Theoretical molecular weight of the macromonomers; ^b^ ref. [23]; ^c^ ref. [26]; ^d^ *N* = Degree of polymerization; ^e^ PDI_MM_ = Polydispersity index of the macromonomers; ^f^ HCT = Hydrophilic collapse temperature; ^g^ DHAT = Dynamic hydrophobic aggregation temperature; ^h^ DRO = Dynamic regime occupation from 5–95 °C (C_6_S_32_) and 5–45 °C (C_11_S_14_) with free (*f*), and the two bound (*b*_1_, *b*_2_) regimes; ^i^ *K*_D,25,*k*_ = dissociation constant [23] of 16-DSA probing polymers *k* at *T =* 25 °C together with the corresponding association constant *K*_A,25,*k*_ and ^j^ *N*_L,*k*_ = number of receptors per polymer determined from Scatchard plots [23] at *T* = 25 °C; ^k^ *a*_iso,*b*_ = isotropic hyperfine coupling constant at *T* = 25 °C as a hydrophobic core polarity index of bound (*b_i_*) spin probes (16-DSA). The lower this *a*_iso,*b*_ value, the less polar the probed environment; ^l^ *a*_iso,*f*_ = isotropic hyperfine coupling constant at *T* = 25 °C for free (*f*) spin probes (16-DSA) in aqueous environment [23].

**Table 2:** Thermodynamic quantities of ln*K*_A,*j*,*k*_ from C_n_S_m_ polymers (*T* = 25 °C).

Quantity	ln*K*_A,*j*,*k*_^a^		
*j* = 25	C_6_S_32_		C_11_S_14_
	(*j* < 30)	(*j* > 50)	(*j* < 45)
∆*G°*_A,25,*k*_ (kJ·mol^−1^)	−25.9 ± 1.1	−28.9 ± 1.2	−31.7 ± 0.4
∆*H°*_A,25,*k*_ (kJ·mol^−1^)	8.6 ± 0.5	−27.1 ± 0.7	17.5 ± 0.2
∆*S°*_A,25,*k*_ (J·mol^−1^·K^−1^)	115.7 ± 1.9	5.8 ± 1.9	165.2 ± 0.7

^a^ Error margins have been determined from propagations of uncertainty according to Equations (S11)–(S14).

**Table 3:** Thermodynamic functions derived from fit functions of both C_n_S_m_ polymers.

C_6_S_32_	C_11_S_14_
$\ln K_{\mathrm{IC},j,6}^{}=\alpha_{1}+\frac{\alpha_{2}}{T}+\frac{\alpha_{3}}{T^{2}}+\frac{\alpha_{4}}{T^{3}}+\frac{\alpha_{5}}{T^{4}}$ (12)	$\ln K_{\mathrm{IC},j,11}^{}=\kappa_{1}\cdot e^{\frac{1}{\kappa_{2}\cdot T}}+\kappa_{3}$ (16)
$\Delta H_{\mathrm{IC},j,6}^{{}^{\circ}}=-R\left(\alpha_{2}+\frac{2\alpha_{3}}{T}+\frac{3\alpha_{4}}{T^{2}}+\frac{4\alpha_{5}}{T^{3}}\right)$ (13)	$\Delta H_{\mathrm{IC},j,11}^{{}^{\circ}}=-\frac{\kappa_{1}R}{\kappa_{2}}\cdot e^{\frac{1}{\kappa_{2}\cdot T}}$ (17)
$\Delta S_{\mathrm{IC},j,6}^{{}^{\circ}}=R\cdot \left(\alpha_{1}-\frac{\alpha_{3}}{T^{2}}-\frac{2\alpha_{4}}{T^{3}}-\frac{3\alpha_{5}}{T^{4}}\right)$ (14)	$\Delta S_{\mathrm{IC},j,11}^{{}^{\circ}}=R\cdot \left(\left(1-\frac{\kappa_{2}}{T}\right)\cdot \kappa_{1}\cdot e^{\frac{1}{\kappa_{2}\cdot T}}+\kappa_{3}\right)$ (18)
$\Delta C_{P,\mathrm{IC},j,6}^{{}^{\circ}}=R\cdot \left(\frac{2\alpha_{3}}{T^{2}}+\frac{6\alpha_{4}}{T^{3}}+\frac{12\alpha_{5}}{T^{4}}\right)$ (15)	$\Delta C_{P,\mathrm{IC},j,11}^{{}^{\circ}}=\frac{\kappa_{1}R}{\kappa_{2}^{2}}\cdot \frac{e^{\frac{1}{\kappa_{2}\cdot T}}}{T^{2}}$ (19)

**Table 4:** Thermodynamic parameters of ln*K*_IC,*j*,*k*_ of C_n_S_m_ polymers at *T* = 25 °C.

	ln*K*_IC,*j*,*k*_	
*j* = 25	C_6_S_32_	C_11_S_14_
∆*G°*_IC,25,*k*_ (kJ/mol)	0.86	−2.87
∆*H°*_IC,25,*k*_ (kJ/mol)	23.12	42.79
∆*S°*_IC,25,*k*_ (J/mol·K)	74.71	153.24
∆*C°*_P*,*IC,25,*k*_ (kJ/mol·K)	1.01	2.86

## Supplementary Materials

Supplementary materials are available online at www.mdpi.com/2073-4360/9/8/324/s1.
