# Supplementary material for: Probing the Nanoscopic Thermodynamic Fingerprint of Paramagnetic Ligands Interacting with Amphiphilic Macromolecules
Source: Polymers (Basel). 2017 Jul 31;9(8):324. doi: 10.3390/polym9080324 (PMC6418530; doi:10.3390/polym9080324)
Supplement: Supplementary file 1 [file polymers-09-00324-s001.pdf]

Supplementary Information

Probing the Nanoscopic Thermodynamic Fingerprint of Paramagnetic Ligands Interacting with Amphiphilic Macromolecules

Jörg Reichenwallner, Christian Schwieger and Dariush Hinderberger*

Institute of Chemistry, Martin-Luther-Universität Halle-Wittenberg, Von-Danckelmann-Platz 4, 06120 Halle, Germany; joerg.reichenwallner@chemie.uni-halle.de (J.R.); christian.schwieger@chemie.uni-halle.de (C.S.)
* Correspondence: dariush.hinderberger@chemie.uni-halle.de; Tel.: +49-345-552-5230

I. Supplementary Figures

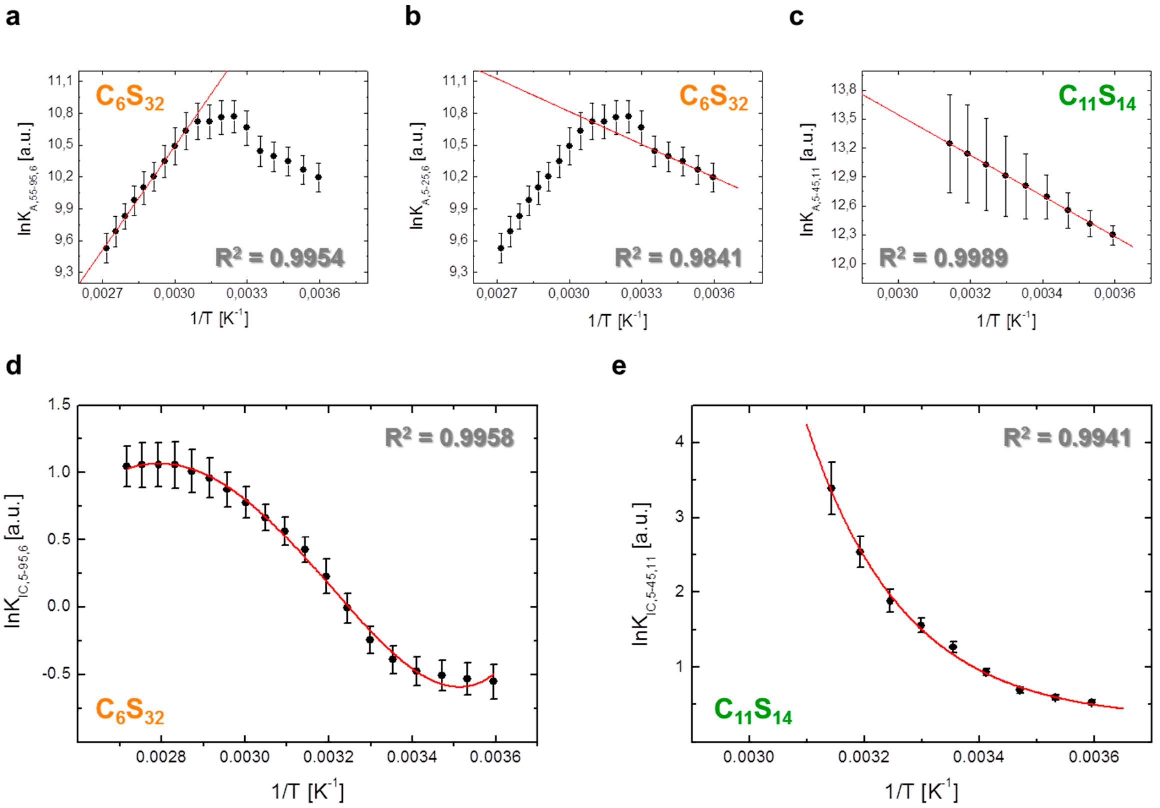

Supplementary Figure S1. Curve fits of $\ln K_{A,j,k}$ and $\ln K_{IC,j,k}$. (**a**) Linear fit of $\ln K_{A,55\text{–}95,6}$ of polymer C_6S_{32} in the temperature range 55 – 95°C; (**b**) Linear fit of $\ln K_{A,5\text{–}25,6}$ of polymer C_6S_{32} in the temperature range 5 – 25°C; (**c**) Linear fit of $\ln K_{A,5\text{–}45,11}$ of polymer $C_{11}S_{14}$ in the temperature range 5 – 45°C; (**d**) Polynomial fit of $\ln K_{IC,5\text{–}95,6}$ of polymer C_6S_{32} in the temperature range 5 – 95°C; (**e**) Exponential fit of $\ln K_{IC,5\text{–}45,11}$ of polymer $C_{11}S_{14}$ in the temperature range 5 – 45°C. Calculated data points and error bars are shown in black, fit curves are shown in red.

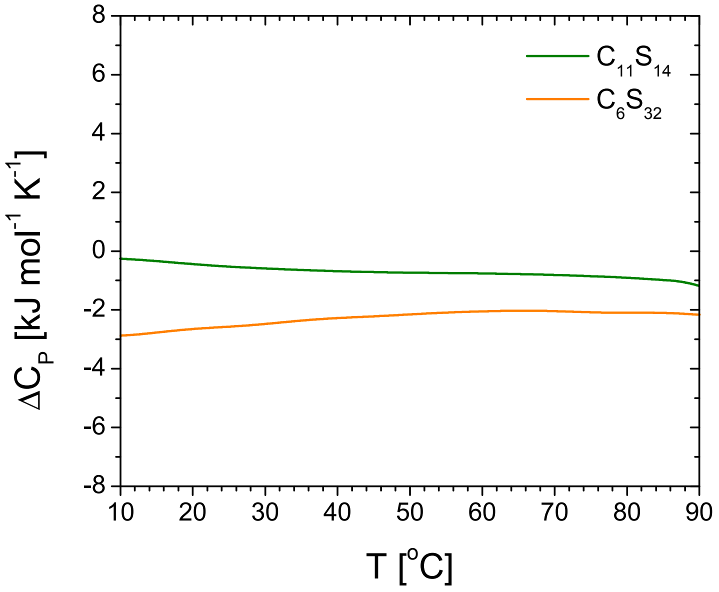

Supplementary Figure S2. DSC thermograms of both polymers loaded with 16-DSA shows the differential heat capacity per mol of macromonomer (ΔC_P) of $C_{11}S_{14}$ (1.66 mM + 29.1 µM 16-DSA, green) and C_6S_{32} (1.78 mM + 18.4 µM 16-DSA, orange). The presented heat capacity traces are corrected for buffer and 16-DSA contributions.

II. Supplementary Tables

Supplementary Table S1. Construction parameters§ for ln$K_{A,j,6}$ and ln$K_{IC,j,6}$ (C_6S_{32}).

j = T [°C]	1/T [K^{-1}]	$[L]_{b1,j,6}$ [µM]	$d[L]_{b1,j,6}$ [µM]	$[L]_{b2,j,6}$ [µM]	$d[L]_{b2,j,6}$ [µM]	$[L]_{f,j,6}$ [µM]	$d[L]_{f,j,6}$ [µM]
**5**	0.003595	97.050	4.563	55.856	4.563	6.714	0.461
**10**	0.003532	96.581	4.318	56.760	4.318	6.279	0.428
**15**	0.003470	96.066	4.080	57.756	4.080	5.798	0.394
**20**	0.003411	94.985	3.882	59.067	3.882	5.568	0.382
**25**	0.003354	92.054	3.847	62.265	3.847	5.301	0.373
**30**	0.003299	87.061	3.869	68.271	3.869	4.287	0.351
**35**	0.003245	78.181	4.207	77.575	4.207	3.864	0.299
**40**	0.003193	69.011	4.846	86.720	4.846	3.890	0.313
**45**	0.003143	61.479	3.586	94.100	3.586	4.041	0.321
**50**	0.003095	56.398	3.839	99.185	3.839	4.037	0.343
**55**	0.003047	52.669	3.426	102.550	3.426	4.401	0.389
**60**	0.003002	48.646	3.836	105.912	3.836	5.062	0.446
**65**	0.002957	45.296	4.161	108.531	4.161	5.792	0.438
**70**	0.002914	42.294	4.531	110.656	4.531	6.670	0.455
**75**	0.002872	40.615	4.705	111.627	4.705	7.377	0.580
**80**	0.002832	39.023	5.011	112.362	5.011	8.236	0.571
**85**	0.002792	38.643	4.705	111.514	4.705	9.463	0.550
**90**	0.002754	38.369	4.729	110.439	4.729	10.811	0.775
**95**	0.002716	38.277	4.266	108.853	4.266	12.491	0.884

§Dynamic regime occupation of 16-DSA for C_6S_{32} polymers. Data sets are adapted from Reichenwallner et al. [1] and are therein depicted in Figure 3b in terms of dynamic fractions $\phi_{i,j,k}$ of regimes f, b_1 and b_2. ($[L]_{b,j,6} = [L]_{b1,j,6} + [L]_{b2,j,6}$; $[L]_{t,6}$ = 159.62 ± 3.88 µM; $c_{P,6}$ = 85 µM).

Supplementary Table S2. Calculated values for ln$K_{A,j,6}$ and ln$K_{IC,j,6}$ (C_6S_{32}).

j = T [°C]	T [K]	1/T [K^{-1}]	$K_{A,j,6}$ [M^{-1}]	ln$K_{A,j,6}$	$K_{IC,j,6}$	ln$K_{IC,j,6}$
**5**	278.15	0.003595	26738.1773	10.1938477	0.57554192	-0.55244321
**10**	283.15	0.003532	28682.6314	10.264047	0.58768882	-0.53155769
**15**	288.15	0.003470	31181.28357	10.3475733	0.60121724	-0.50879895
**20**	293.15	0.003411	32527.45264	10.3898397	0.62185703	-0.47504507
**25**	298.15	0.003354	34232.21309	10.4409224	0.67639182	-0.39098275
**30**	303.15	0.003299	42657.67501	10.6609625	0.7841758	-0.24312205
**35**	308.15	0.003245	47483.7895	10.7681437	0.99223804	-0.00779224
**40**	313.15	0.003193	47158.13742	10.7612619	1.25661632	0.22842265
**45**	318.15	0.003143	45337.57137	10.7218914	1.53061468	0.42566941
**50**	323.15	0.003095	45390.71523	10.7230629	1.75864438	0.56454327
**55**	328.15	0.003047	41515.63048	10.6338253	1.94706588	0.66632356
**60**	333.15	0.003002	35917.08704	10.4889684	2.17718103	0.77803093
**65**	338.15	0.002957	31211.30428	10.3485356	2.39604086	0.87381773
**70**	343.15	0.002914	26920.76254	10.2006531	2.61633218	0.96177341
**75**	348.15	0.002872	24207.88572	10.0944337	2.74842361	1.01102751
**80**	353.15	0.002832	21541.23541	9.9777243	2.87940493	1.05758365
**85**	358.15	0.002792	18569.34422	9.82926734	2.88575305	1.05978589
**90**	363.15	0.002754	16081.95687	9.68545323	2.87831148	1.05720383
**95**	368.15	0.002716	13735.66522	9.52775103	2.84383134	1.04515221

Supplementary Table S3. Construction parameters§ for ln$K_{A,j,11}$ and ln$K_{IC,j,11}$ ($C_{11}S_{14}$).

j = T [°C]	1/T [K^{-1}]	$[L]_{b1,j,11}$ [µM]	$d[L]_{b1,j,11}$ [µM]	$[L]_{b2,j,11}$ [µM]	$d[L]_{b2,j,11}$ [µM]	$[L]_{f,j,11}$ [µM]	$d[L]_{f,j,11}$ [µM]
**5**	0.003595	75.512	1.537	127.743	1.761	0.915	0.047
**10**	0.003532	72.550	1.648	130.806	1.642	0.813	0.054
**15**	0.003470	67.907	1.857	135.555	1.475	0.708	0.065
**20**	0.003411	57.633	2.204	145.920	1.254	0.617	0.070
**25**	0.003354	44.828	2.824	158.791	1.028	0.552	0.092
**30**	0.003299	35.426	3.276	168.246	0.885	0.497	0.103
**35**	0.003245	26.929	3.918	176.799	0.776	0.442	0.106
**40**	0.003193	14.951	3.081	188.825	0.604	0.394	0.101
**45**	0.003143	6.642	2.278	197.175	1.217	0.354	0.090
**50**	0.003095	-	-	204.170	-	-	-
**55**	0.003047	-	-	204.170	-	-	-
**60**	0.003002	-	-	204.170	-	-	-
**65**	0.002957	-	-	204.170	-	-	-
**70**	0.002914	-	-	204.170	-	-	-
**75**	0.002872	-	-	204.170	-	-	-
**80**	0.002832	-	-	204.170	-	-	-
**85**	0.002792	-	-	204.170	-	-	-
**90**	0.002754	-	-	204.170	-	-	-
**95**	0.002716	-	-	204.170	-	-	-

§Dynamic regime occupation of 16-DSA for $C_{11}S_{14}$ polymers. Data sets are adapted from Reichenwallner et al. [1] and are therein depicted in Figure 3c in terms of dynamic fractions $\phi_{i,j,k}$ of regimes f, b_1 and b_2. ($[L]_{b,j,11} = [L]_{b1,j,11} + [L]_{b2,j,11}$; $[L]_{t,11}$ = 204.17 ± 5.93 µM; $c_{P,11}$ = 622 µM).

Supplementary Table S4. Calculated values of ln$K_{A,j,11}$ and ln$K_{IC,j,11}$ ($C_{11}S_{14}$).

j = T [°C]	1/T [K^{-1}]	$K_{A,j,11}$ [M^{-1}]	ln$K_{A,j,11}$	$K_{IC,j,11}$	ln$K_{IC,j,11}$
**5**	0.003595	218597.815	12.2949889	1.69168582	0.52572556
**10**	0.003532	246311.7562	12.4143533	1.80297743	0.58943943
**15**	0.003470	283085.8863	12.5535056	1.9961816	0.69123616
**20**	0.003411	324635.6145	12.6904586	2.53188324	0.92896339
**25**	0.003354	363193.926	12.8026922	3.54226635	1.26476673
**30**	0.003299	403281.2895	12.9073896	4.74929399	1.55799597
**35**	0.003245	454330.1029	13.0265793	6.56547353	1.88182463
**40**	0.003193	508793.5671	13.1397976	12.6292503	2.53601558
**45**	0.003143	566698.6429	13.2475829	29.6876729	3.39073191

Supplementary Table S5. Fit parameters $a_{j,k}$ and $b_{j,k}$ for $\ln K_{A,j,k}$.

**Polymer *k***	***T* range [°C]**	**R^2**	**$a_{j,k} \pm \Delta a_{j,k}$**	**$b_{j,k} \pm \Delta b_{j,k}$**
**C_6S_{32}**	≤ 25	0.98411	13.90997 ± 0.22731	–1031.69593 ± 65.41604
	≥ 55	0.99540	0.69999 ± 0.22443	3264.23482 ± 78.46214
**$C_{11}S_{14}$**	5 – 45	0.99894	19.87061 ± 0.08536	–2108.28452 ± 24.31021

Supplementary Table S6. Fit parameters α_x and κ_y for $\ln K_{IC,j,k}$.

**Polymer *k***	***T* range [°C]**	**fit**	**R^2**	***x***	***y***	**α_x**	**κ_y**
**C_6S_{32}**	5 – 95	Poly.	0.99407	1		303.78682	–
				2		–473108.6302	–
				3		$2.68781 \cdot 10^{8}$	–
				4		$-6.60985 \cdot 10^{10}$	–
				5		$5.95024 \cdot 10^{12}$	–
**$C_{11}S_{14}$**	5 – 45	Exp.	0.99587		1	–	$3.819 \cdot 10^{8}$
					2	–	$-1.68608 \cdot 10^{-4}$
					3	–	0.29148

III. Supplementary Methods

A. Explicit Derivation of Equation (6) of the main manuscript.

The combination of a receptor R with ligand L towards a receptor-ligand complex RL is best described by the chemical equation [2]:

$$\mathrm{R}+\mathrm{L} \underset{k_d}{\overset{k_a}{\rightleftharpoons}} \mathrm{RL} \quad . \tag{S1}$$

The reaction rate constants facilitate the description of either ligand association (k_a) or dissociation (k_d). The total receptor concentration $[\mathrm{R}]_{t,k}$ and the total ligand concentration $[\mathrm{L}]_{t,k}$ are described as:

$$[\mathrm{R}]_{t,k} = [\mathrm{RL}]_{j,k} + [\mathrm{R}]_{f,j,k} \tag{S2}$$

$$[\mathrm{L}]_{t,k} = [\mathrm{RL}]_{j,k} + [\mathrm{L}]_{f,j,k} \tag{S3}$$

where $[\mathrm{RL}]_{j,k}$ is the ligand-receptor complex concentration, $[\mathrm{R}]_{f,j,k}$ is the concentration of vacant receptors and $[\mathrm{L}]_{f,j,k}$ is the concentration of free ligand. The law of mass action gives an equilibrium association constant $K_{\mathrm{A},j,k}$ by the relation:

$$K_{\mathrm{A},j,k} = \frac{\prod [x]_{\mathrm{p},i,j,k}}{\prod [x]_{\mathrm{e},i,j,k}} = \frac{[\mathrm{RL}]_{j,k}}{[\mathrm{R}]_{f,j,k}[\mathrm{L}]_{f,j,k}} \tag{S4}$$

The product $[x]_{\mathrm{p},i,j,k}$ can be considered as the molecular complex $[\mathrm{RL}]_{j,k}$ when educts $[x]_{\mathrm{e},i,j,k}$ described as $[\mathrm{R}]_{f,j,k}$ and $[\mathrm{L}]_{f,j,k}$ associate. With equation (S2) and (S3), equation (S4) can be rewritten for the case of tight binding interactions where $[\mathrm{L}]_{f,j,k} << [\mathrm{L}]_{t,k}$ and the value of the dissociation constant $K_{\mathrm{D},j,k}$ is in the range of the total receptor concentration ($[\mathrm{R}]_{t,k} \approx K_{\mathrm{D},j,k} = K_{\mathrm{A},j,k}^{-1}$). Therefore, the $K_{\mathrm{A},j,k}$ value is initially expressed as [2]:

$$K_{\mathrm{A},j,k} = \frac{[\mathrm{RL}]_{j,k}}{([\mathrm{R}]_{t,k} - [\mathrm{RL}]_{j,k}) \cdot ([\mathrm{L}]_{t,k} - [\mathrm{RL}]_{j,k})} \quad . \tag{S5}$$

The receptor-ligand complex concentration $[\mathrm{RL}]_{j,k}$ is now termed as the bound fraction of ligand $[\mathrm{L}]_{b,j,k}$. For this derivation no differentiation between Brownian and free diffusion of ligand has to be considered. Generally, $[\mathrm{L}]_{b,j,k}$ is the sum of $[\mathrm{L}]_{b1,j,k}$ and $[\mathrm{L}]_{b2,j,k}$. The total receptor concentration $[\mathrm{R}]_{t,k}$ is the product of the polymer concentration $c_{\mathrm{P},k}$ and the number of ligand binding sites $N_{\mathrm{L},k}$ so that:

$$[\mathrm{R}]_{t,k} = N_{\mathrm{L},k} c_{\mathrm{P},k} \quad . \tag{S6}$$

Collecting the aforementioned assumptions, equation (S5) can be rewritten as:

$$K_{\mathrm{A},j,k} = \frac{[\mathrm{L}]_{b,j,k}}{(N_{\mathrm{L},k}c_{\mathrm{P},k} - [\mathrm{L}]_{b,j,k}) \cdot ([\mathrm{L}]_{t,k} - [\mathrm{L}]_{b,j,k})} = \frac{1}{\left(\frac{N_{\mathrm{L},k}c_{\mathrm{P},k}}{[\mathrm{L}]_{b,j,k}} - 1\right) \cdot \left(\frac{[\mathrm{L}]_{t,k}}{[\mathrm{L}]_{b,j,k}} - 1\right)} \quad \text{(S7)}$$

Expanding the right-hand side of equation (S7) leads to the expression:

$$K_{\mathrm{A},j,k} = \frac{1}{\left(\frac{N_{\mathrm{L},k}c_{\mathrm{P},k}[\mathrm{L}]_{t,k}}{[\mathrm{L}]_{b,j,k}} - N_{\mathrm{L},k}c_{\mathrm{P},k} - [\mathrm{L}]_{t,k} + [\mathrm{L}]_{b,j,k}\right)} \quad \text{(S8)}$$

that further simplifies into:

$$K_{\mathrm{A},j,k} = \frac{1}{\left(\frac{N_{\mathrm{L},k}c_{\mathrm{P},k}[\mathrm{L}]_{t,k}}{[\mathrm{L}]_{b,j,k}} - N_{\mathrm{L},k}c_{\mathrm{P},k} - [\mathrm{L}]_{f,j,k}\right)} = \frac{1}{\left([\mathrm{R}]_{t,k}\left(\frac{[\mathrm{L}]_{t,k}}{[\mathrm{L}]_{b,j,k}} - 1\right) - [\mathrm{L}]_{f,j,k}\right)} \quad \text{(S9)}$$

when applying equation (S3) in the form of $[\mathrm{L}]_{t,k} = [\mathrm{L}]_{b,j,k} + [\mathrm{L}]_{f,j,k}$ (this expression in equation (S9) corresponds to equation (6) in the main manuscript). The $[\mathrm{R}]_{t,k}$ value can be calculated from the product of $N_{\mathrm{L},k}$ as determined from according Scatchard plots [1] and $c_{\mathrm{P},k}$ that is already known from sample preparation (see Table 1). The total ligand concentration $[\mathrm{L}]_{t,k}$ is obtained from double integration and therefore $[\mathrm{L}]_{f,j,k}$ as well as $[\mathrm{L}]_{b,j,k}$ can be obtained from the fractions $\phi_{i,j,k}$ emerging from EPR spectral simulations in the form $\phi_{i,j,k} \cdot [\mathrm{L}]_{t,k}$ as described in Reichenwallner et al. [1].

B. Curve fits of ln$K_{A,j,k}$ and ln$K_{IC,j,k}$.

The original curve fits from equations (8) (Supplementary Figure S1a–c), (12) (Supplementary Figure S1d) and (16) (Supplementary Figure S1e) of the main manuscript are shown as the basis for all consequent thermodynamic analyses in the main text.

Fit parameters for ln$K_{A,j,k}$ from equation (8).

The linear regression of decisive parts from the van´t Hoff plots of ln$K_{A,j,k}$ in Figure 3a and Supplementary Figure S1a–c was conducted with following equation:

$$y_{j,k} = a_{j,k} + b_{j,k} \cdot x = \frac{\Delta S^\circ_{A,j,k}}{R} - \frac{\Delta H^\circ_{A,j,k}}{R} \cdot \frac{1}{T} = \ln K_{A,j,k} \quad \text{(S10)}$$

so that $\Delta H^\circ_{A,j,k} = -R \cdot b_{j,k}$ and $\Delta S^\circ_{A,j,k} = R \cdot a_{j,k}$ where R is the universal gas constant. The fit parameters $p_1 = a_{j,k}$ and $p_2 = b_{j,k}$ together with their errors $\Delta p_1 = \Delta a_{j,k}$ and $\Delta p_2 = \Delta b_{j,k}$ are given in Supplementary Table S5 and error limits for thermodynamic quantities θ are calculated according to the propagation of uncertainty:

$$\Delta\theta = \sum_i \left| \frac{\partial \theta}{\partial p_i} \right| \cdot \Delta p_i \quad \text{(S11)}$$

so that:

$$\Delta\Delta H^\circ_{A,j,k} = -\Delta b_{j,k} R \quad \text{(S12)}$$

$$\Delta\Delta S^\circ_{A,j,k} = \Delta a_{j,k} R \quad \text{(S13)}$$

$$\Delta\Delta G^\circ_{A,j,k} = R \cdot \left(\Delta b_{j,k} + T \Delta a_{j,k} \right) \quad . \quad \text{(S14)}$$

Fit parameters for ln$K_{IC,j,k}$ from equations (12) and (16).

The polynomial (equation (12)) and exponential (equation (16)) curve regression of the van´t Hoff plots of ln$K_{IC,j,k}$ in Figure 3b and Supplementary Figure S1d–e fit values are shown in Supplementary Table S6. Reliable error limits could not be determined from the applied multiparameter non-linear curve fits.

IV. Supplementary References

[1] Reichenwallner, J.; Thomas, A.; Nuhn, L.; Johann, T.; Meister, A.; Frey, H.; Hinderberger, D. Tunable dynamic hydrophobic attachment of guest molecules in amphiphilic core-shell polymers. *Polym. Chem.* **2016**, *7*, 5783–5798.

[2] Copeland, R.A. *Enzymes: A Practical Introduction to Structure, Mechanism and Data*, 2nd ed.; John Wiley & Sons: Hoboken, NJ, USA, 2000.
